# What’s a book club doing at a medical conference?

**DOI:** 10.15694/mep.2018.0000146.1

**Published:** 2018-07-18

**Authors:** Margaret Chisolm, Amin Azzam, Manasa Ayyala, Rachel Levine, Scott Wright

**Affiliations:** 1 Johns Hopkins University School of Medicine; 2 University of California; 3 Rutgers New Jersey Medical School; 4 Johns Hopkins University School of Medicine

**Keywords:** medical education: CME, medical humanities, self-care, physician satisfaction, continuing professional development

## Abstract

This article was migrated. The article was marked as recommended.

The prevalence of burnout among physicians in the United States now exceeds 50%, with many physicians feeling isolated and lacking in a sense of community. Book clubs among colleagues may be one way to foster community and restore joy to medicine. The authors introduced two book clubs at the annual meetings of the Society for General Internal Medicine (SGIM) and the Association for Academic Psychiatry (AAP). Response rates for completed surveys for the SGIM and AAP book clubs were 71% and 86%, respectively. About half of the book club participants were already members of a book club, and had read an average of 10 non-medical books in the past year. Eighty-one percent reported the discussions had “a lot” or “tremendous” impact on their learning, and that they would be “likely” or “very likely” to look for a non-medical book in the future as a resource to assist in their professional growth. Sixty-seven percent said they would be “likely” or “very likely” to organize their own book club. Participants listed the “most enjoyable and/or impactful non-medical book read in past year.” Survey responses speak to the impact of book club participation on attendees’ professional growth, learning, and recognition of overall value of reading non-medical books. These findings support the role of the humanities in professional development to encourage physicians to challenge assumptions, tolerate ambiguity, appreciate cultural influences, and honor the unique perspectives of our patients. In the increasingly complex and challenging work environment of academic health centers, faculty must find mechanisms to maintain workplace meaning and prevent burnout. Reading a book prior to attending one’s annual professional society meeting and participating in thoughtful discussions was enjoyable and useful. When facilitators are thoughtfully prepared to guide conversations, professional growth can result in useful insights related to academic practices and pursuits.

## The Problem

Practicing medicine in the 21 ^st^ century is challenging, as changes in health care delivery systems have resulted in increasing physician workloads, productivity targets, electronic health record clerical demands, and regulatory requirements ( Shanafelt *et al.*, 2015). An explosion of scientific information and perpetually evolving changes in practice guidelines have added to these burdens. The prevalence of burnout among physicians in the United States now exceeds 50% ( Shanafelt *et al.,* 2015), with many physicians feeling isolated and lacking in a sense of community. Bringing joy back to medicine is critical but best practices for how to do this remain unclear.

Book clubs among colleagues may be one way to foster community and restore joy to medicine. These gatherings bring people together and promote the exchange of ideas and opinions. Sitting together, sharing a common experience, and discussing lessons learned and implications for medical practice may reduce feelings of loneliness at work and promote connectedness. While reading medical journals is essential for keeping up to date, reading books from non-scientific fields can support professional growth in uniquely valuable ways. Titles drawn from the humanities can help adult learners appreciate different perspectives, deepen reflection, and nourish humanistic values in medicine ( Kidd and Castano, 2013).

Many physicians have not been exposed to a book club in a local professional setting, and those who have experienced one in the past may now have too many other demands on their time to coordinate one. Consequently we decided to organize book clubs for these busy academic clinicians. Scheduling the book clubs during annual medical conferences creates an opportunity for healthcare professionals to appreciate the value of the book club experience at a time when they have already set aside time, away from their daily routine. In seeing the benefit from their involvement, we hoped participants might become book club champions to recreate these focused sessions at their home institutions.

## What We Did

The authors introduced two book clubs - one for general internists and another for psychiatrists - at two specialty medical conferences: the Society for General Internal Medicine (SGIM) and the Association for Academic Psychiatry (AAP). Approximately 2500 general internal medicine residents, fellows, and practitioners attend the SGIM meeting each year. The book club was first introduced at the 2016 SGIM annual meeting. After the anecdotal success of that first session, we committed to repeating the session - with new book selections - the following year at both the SGIM and AAP annual meetings and designing a formal evaluation. Approximately 300 general psychiatry residents, psychiatry fellows, and psychiatrists attend the AAP meeting, where the book club debuted in 2017.

### SGIM Book Club

For the 2017 SGIM book club, authors MA, RL and SW selected the book “Wonder” by R.J. Palacio. This book selection was inspired by the meeting’s theme, ‘Resilience & Grit.’ The SGIM book club was announced in the annual meeting’s program agenda more than 4 months before the conference to give interested attendees time to read the book (see link to book club description: https://connect.sgim.org/sgim17/program/bookclub). To encourage deeper dialogue and interactions at the book club, space was limited to 40 registrants. The session was designed to be interactive starting with an exercise for participants to get to know one another and practice reflection. Then, a guided discussion was directed to select themes from the book (most notably empathy, asking for help, and perspective taking) focused on attendees’ personal stories of how these concepts were relevant to their work in patient care, education, research, and leadership.

### AAP Book Club

For the 2017 AAP book club, authors AA and MC selected Alison Bechdel’s memoir “ Fun Home: A Family Tragicomic”. MC, who has been leading the Reading the Mind Book Group for psychiatry residents at Johns Hopkins University School of Medicine since 2013 ( Kan *et al.*, 2015; Lal *et al.*, 2016), noted that this had been one of that group’s favorite selections. The AAP book club was dubbed the “Presidential Book Club” (in honor of author AA, then President of the organization) and was highlighted on the meeting registration website, where attendees could sign up to participate. The book club was scheduled into a “prime time” slot following the first afternoon of the meeting, and immediately prior to the opening welcome reception. In 2017, 21 AAP attendees participated in the inaugural meeting of the book club. The soundtrack of the “Fun Home” Broadway show was playing in the background, and free adult beverages were served as attendees arrived. Suggested discussion questions, posted previously on the meeting website, were available in print form at the book club meeting to promote dialogue, which AA and MC facilitated. (Example of one of the discussion questions: “Bechdel has obsessive-compulsive symptoms, which began with a particularly intense onset at age 10. How does her OCD contribute to the documentation of her childhood?”).

## What We Found

The authors surveyed 2017 SGIM and AAP book club attendees following the respective book club sessions. The authors distributed the SGIM survey to book club attendees via email while the AAP survey was distributed and collected at the meeting. The Johns Hopkins University School of Medicine institutional review board designated the project as exempt from further review.

The SGIM and AAP surveys were identical, with the exception of the book titles. Each survey contained 13 items organized around 3 categories: reading preferences and history, perspectives related to participating in the meeting’s book club, and future reading plans. The survey contained a mix of quantitative and qualitative assessment items. Response rates for completed surveys for the SGIM and AAP book clubs were 71% (24/34; email addresses were unknown for 6 participants) and 86% (18/21), respectively.

As seen in Table 1, about half of the SGIM and AAP book club participants were already members of a book club, and had read an average of 10 non-medical books in the past year. Eighty-one percent reported that the SGIM and AAP book Ccub discussions had “a lot” or “tremendous” impact on their learning. An equal percentage from both meetings (81%) reported that they would be “likely” or “very likely” to look for a non-medical book in the future as a resource to assist in their professional growth, with 67% saying they would be “likely” or “very likely” to organize a book club of their own.

**Table 1.** Quantitative Item Responses to Survey Questions from the 42 Participants in the Book Clubs

Item	SGIM N = 24	AAP N = 18
***Reading Books***		
Current book club member	67%	39%
Mean (range) number of non-medical books “read” in past 12 months	9.6 (0-20)	11.2 (0-42)
***The SGIM/AAP Book Clubs ^1^ ***		
% endorsing “a lot” or “tremendous”		
Impact of discussion on learning	86%	75%
Impact of reading and discussion on recognition of professional value of reading non-medical books	69%	53%
Impact of reading Book Club selection on professional growth	62%	35%
Impact of reading the selection on clinical practice	36%	25%
***Future Reading Plans ^2^ ***		
% “likely” or “very likely” to look for nonmedical book as a resource to assist in professional growth	86%	75%
% “likely” or “very likely” to, if not already, set up book club (personal or professional)	77%	57%
Mean (range) number of non-medical books planned to be read in next 12 months	12.6 (4-24)	12.3 (0-45)

^1^ Based on 5 point scale Likert responses 1=none, 2=a little, 3=a lot, 4=tremendous

^2^ Based on 5 point Likert responses 1=very unlikely, 2=unlikely, 3=likely, 4=very likely

From the open-ended questions and qualitative comments, the insights and perceptions were fairly consistent across individuals and between the two book clubs. The following 4 examples are representative of the sentiments that were shared:

*“I was surprised to see the clear links to medical practice- not something I had appreciated before that session.” (SGIM participant)*

*“What a wonderful session to go to. We shared life lessons that can be applicable in our practices and personal lives. It was refreshing for the soul to spend a session the way we did. And how impactful to have our national organization place importance (not just in words, but in deeds) on the promotion of self-care like this.” (SGIM participant)*

*“Discussion was crucial - really broadened my understanding.” (AAP participant)*

*“Great book and very relevant to deepening cultural understanding and empathy.” (AAP participant)*

A selection of book titles described by participants as “most enjoyable and/or impactful non-medical book read in past year” is shown in Table 2.

**Table 2.** Selected “Reading Books” Item Responses

Most enjoyable non-medical book read in past year	Most impactful non-medical book read in past year
What Alice Forgot The Handmaid’s Tale ^1, 2^ The Nightingale Dreamland The Chinese Chef The Night Manager The Buried Giant Little Bee Transatlantic Hamilton A Man Called Ove A Gentleman in Moscow The Lowlands The Harvest Sherlock Holmes I’m Just a Person ^3^ My Struggle series **Fun Home** ^2^ Brain on Fire Tokyo Ghoul Brave New World Americanah Big Little Lies Redshirts Unselfie Ready Player One The Yipping Tiger Positive Discipline The Valley of Amazement The Name of the Wind	Killing Lincoln Dreamland ^3^ Extreme Ownership Natty Professor Weapons of Math Destruction Blink I Know How She Does It Just Mercy Superbosses Visual Intelligence A Gentleman in Moscow ^3^ Stoner The Highly Sensitive Person **Wonder** I’m Just a Person ^3^ Are You My Mother? The Bluest Eye

^1^ named by both an SGIM and an AAP respondent

^2^ named by more than one respondent

^3^ named as both most enjoyable and impactful

## Lessons Learned

Despite navigating busy careers in academic general internal medicine and psychiatry, participants in the SGIM and AAP book clubs report being avid readers of non-medical books. The SGIM and AAP book club participants offered robust lists of books that had impacted them and that they had enjoyed reading in the past year; the limited overlap of titles across individuals and between the SGIM and AAP groups speaks to the distinctive and personalized approaches that are at play in selecting books to read for pleasure. Nonetheless, the SGIM/AAP book club selections appeared on both club’s participants’ individual lists as the most enjoyable and/or impactful non-medical book read in past year, suggesting the appropriateness of the choices and the value of the reflections spawned by the readings and discussions ( Schön,1987).

The responses to both the quantitative and open-ended qualitative question speak to the impact of the SGIM and AAP book club participation on attendees’ professional growth, learning, and recognition of overall value of reading non-medical books. These findings support the role of the humanities in professional development to encourage physicians to challenge assumptions, tolerate ambiguity, appreciate cultural influences, and honor the unique perspectives of our patients ( Sklar, 2017).

Several limitations of this project and the results are worth considering. First, because book clubs represent intimate discussion groups, the sample size at both meetings was small. Second, inevitable selection bias exists as book readers are far more likely to sign up to attend a book club at a national meeting than are non-book readers. Thus, those who proactively secured the book, read it, and showed up for the book club discussion are not representative of all members of the societies. This limitation can also be a strength as society members who attend such a book club are likely to appreciate the benefits of non-medical reading and the opportunity to engage with like-minded colleagues in a structured professional setting with the aim of enhancing joy in medicine through collegiality and community. Third, we relied on self-report measures to learn about the value and impact of the book club. Finally, these findings from internal medicine and psychiatry may not be generalizable to other medical specialties. Nevertheless, the authors are hopeful that the SGIM and AAP book clubs will continue at the annual meetings of these societies to allow for further participant reflection and systematic research on impact.

## Take Home Messages

• In the increasingly complex and challenging work environment of academic health centers, it is imperative that faculty find mechanisms to maintain workplace meaning and prevent burnout.
• Reading a book prior to one’s annual professional society meeting and then participating in thoughtful discussions among peers was judged to be enjoyable and useful.
• When facilitators are thoughtfully prepared to guide conversations, professional growth can result in useful insights related to academic practices and pursuits.

## Notes On Contributors

Dr. Chisolm is Associate Professor and Vice Chair for Education in the Department of Psychiatry and Behavioral Sciences at Johns Hopkins. She is a leading public and academic proponent of using social media to improve clinical care. Along with Dr. Wright and others, she is co-developer of ww.closler.org dedicated to clinical excellence.

Dr. Azzam is a Full Clinical Professor at University of California, San Francisco, School of Medicine, Department of Psychiatry; University of California, Berkeley, School of Public Health; and Samuel Merritt University, Health Sciences Simulation Center. He focuses on interprofessional, simulation-based, and educational innovation.

Dr. Ayyala is Assistant Professor in the Division of General Internal Medicine at Rutgers New Jersey Medical School. She is committed to the education of medical students and trainees, and focuses on promoting physician wellness, professional development, and health equity.

Dr. Levine is Associate Professor of Medicine at the Johns Hopkins School of Medicine. She spends the majority of her time coaching faculty to improve their teaching skills and in achieving academic success in her role as Associate Dean for Faculty Educational Development in the School of Medicine.

Dr. Wright is Anne Gaines and G. Thomas Miller Professor of Medicine at Johns Hopkins. He is Chief of the Department of General Internal Medicine at Johns Hopkins Bayview Medical Center and Director of the Miller Coulson Academy of Clinical Excellence, committed to recognizing and promoting excellence in patient care.
