# Supplementary material for: What’s a book club doing at a medical conference?
Source: MedEdPublish (2016). 2018 Jul 18;7:146. [Version 1] doi: 10.15694/mep.2018.0000146.1 (PMC10701802; doi:10.15694/mep.2018.0000146.1)
Supplement: Peer review history

# Reviewer report: Reviewer response for version 1

**DOI:** 10.21956/mep.19622.r29187

**Published:** 2019-07-20

**Authors:** Gary D. Rogers

**Affiliations:** 1 Griffith University

This review has been migrated. The reviewer awarded 4 stars out of 5 Thanks, colleagues, for sharing this interesting study on a novel idea for the continuing professional development of health professionals. As you have pointed out, as an optional activity, the intervention is, sadly, likely to be taken up by those who, in some senses, ‘need it least’ as they are probably already reading recreationally and are reflectively inclined. It is not easy to see how this effect could be overcome in the post registration space but for health professional students any activities like this really should be compulsory and assessed if we truly believe them to be important. Tools for assessing the affective learning associated with emotionally impactive activities like this are now available. My only quibble with the paper is that it would have been nice to see the authors utilise a deeper qualitative analysis method, and perhaps even participant interviews, since the learning from this kind of activity is complex and nuanced, which would have lent itself to a qualitative methodology like interpretative phenomenological analysis.

Reviewer Expertise:

NA

No decision status is available

# Reviewer report: Reviewer response for version 1

**DOI:** 10.21956/mep.19622.r29193

**Published:** 2018-08-23

**Authors:** Jonathan McFarland

**Affiliations:** 1 Sechenov University, Moscow

This review has been migrated. The reviewer awarded 5 stars out of 5 What a simple but brilliant idea, and one taken directly from Osler's teachings. We are continuously debating on how to reintroduce the humanities into medical education and thus practice but seldom do we touch on the "how". This article's great interest lies in the fact that it can be readily introduced around the world at different medical schools and conferences, and I, for one, am really looking forward to discussing these options. Maybe we could introduce this at the next AMEE conference? I would be very happy to work on this idea if it is deemed appropriate.

Reviewer Expertise:

NA

No decision status is available

# Reviewer report: Reviewer response for version 1

**DOI:** 10.21956/mep.19622.r29186

**Published:** 2018-08-03

**Authors:** Irina Markovina

**Affiliations:** 1 Sechenov First Moscow State Medical University (Sechenov University)

This review has been migrated. The reviewer awarded 5 stars out of 5 I would like to point out that I enjoyed reading the article written by the Johns Hopkins University team. It discusses the experience that could be referred to as one of the best practices that should be repeated and even further developed by other medical institutions.I would like to focus on two points. First. I find the idea of the authors very interesting and promising. To treat burnout by reading non-medical books and what is even more important, by discussing the stories read is an excellent idea. Reading books is a kind of activity that globally goes out of fashion nowadays. However, as long as the medical profession retain this skill, patients have a chance of being listened to, heard and understood. Moreover, talking what has been read over definitely contributes to developing or rather “keeping in good shape” the communication skills of the medical professionals and “fosters the sense of community”, as the authors say. Second, I find the research itself well designed and conducted. I read the Lessons Leaned section with great interest. Compiling the list of books “for fun” and for “food for thought” is also an excellent idea. Take Home Message section is informative and convincing. I completely agree with the Johns Hopkins University team that their findings prove the role of the humanities, and literature in particular, in the professional development of physicians. The role that is, to my mind, underestimated and not generally recognized. The article is a valuable contribution to changing the present situation. I think we have to try to implement the idea of a book club in the Russian medical university setting.

Reviewer Expertise:

NA

No decision status is available

# Reviewer report: Reviewer response for version 1

**DOI:** 10.21956/mep.19622.r29190

**Published:** 2018-07-24

**Authors:** Trevor Gibbs

**Affiliations:** 1 AMEE

This review has been migrated. The reviewer awarded 5 stars out of 5 A very interesting and stimulating paper to read and one that looks tangentially at how we educate our future and present doctors. One issue that seems to have been ever-present is that doctors seldom leave the busy world of healthcare to look and learn from the wider and real world of literature. Just as paintings can covey a deep and meaningful message to those who view it, a book can provide similar insightful thoughts and eventual discussions. I would agree with my co-reviewers that this paper should be recommended to all curriculum designers, particular those who are prepared to think out of the box and introduce newer and exciting ways of learning about life.

Reviewer Expertise:

NA

No decision status is available

# Reviewer report: Reviewer response for version 1

**DOI:** 10.21956/mep.19622.r29189

**Published:** 2018-07-22

**Authors:** Sofia Belogubova

**Affiliations:** 1 Sechenov University

This review has been migrated. The reviewer awarded 5 stars out of 5 I found this article quite useful and up-to-date containing current information on the theme of humanities in medicine. In my opinion, this valuable field provides deep a understanding of the patients’ conditions, their ideas, concerns, expectations, and wishes about their medical life stories. There is no doubt that reading books and discussing them amongst ourselves may help the humanities fill in the gaps in medicine practice, but we should never forget about music, theatre, paintings and other pieces of art which cover all human interests and their individualities. The authors of this article made the significant conclusion that further research is needed as the limitation of a non-representative sample, and the difficulty of generalizability and the outcomes cannot be definitely reliable. The humanities in the 21st century is such a diverse field, for instance, virtual book clubs do exist and a considerable number of doctors are members of this club ,where they discuss and reflect on books online at http://doctorsbookclub.com. Hopefully, this virtual platform may let the researchers continue their investigations. It would be really great if modern hospitals and medical schools could introduce book clubs, music and theatre clubs, maybe even poetry evenings. It is of vital importance to provide doctors and students with an opportunity to cope with burnout and the loss of meaning in such an honorable and grateful profession.

Reviewer Expertise:

NA

No decision status is available

# Reviewer report: Reviewer response for version 1

**DOI:** 10.21956/mep.19622.r29192

**Published:** 2018-07-20

**Authors:** Elena Pastor-Ramon

**Affiliations:** 1 Virtual Health Sciences Library of the Balearic Islands

This review has been migrated. The reviewer awarded 5 stars out of 5 I enjoyed reading this article very much. Sometimes we do not see physicians as readers , just as healthcare professionals, I am librarian who has been working for more than 18 years in healthcare, so books are my passion and my work, so, knowing that there are these kinds of initiatives at medical conference, I think is fantastic.One of the main mistakes that we make when we start our professional life is to stop reading or just read in particular periods, especially on holidays, I think this is a big mistake especially among health professionals from all disciplines. There is an enormous amount of feelings and burnout against which you have to struggle: death, bad news, patient-professional relationship, etc. Reading a book not related with our work makes us break free from these kinds of situations and teach us how to face different kinds of situations.I have read some of the books that the club proposed, and I think they are perfect to understand that the patient and their family are not just the people who go to the consultation room or lie on the operating table: they have a history, they have troubles, and you need to see beyond the moment of the consultation. These are books that present some of the more common situations that anybody could find in their lives.If I may I would like to recommend Marian Keyes books’, maybe you could think they are books that are just romantic, but she talks about some personal situations such as depression or alcoholism, and deals with issues related to gender violence.

Reviewer Expertise:

NA

No decision status is available

# Reviewer report: Reviewer response for version 1

**DOI:** 10.21956/mep.19622.r29188

**Published:** 2018-07-18

**Authors:** P Ravi Shankar

**Affiliations:** 1 American International Medical University

This review has been migrated. The reviewer awarded 4 stars out of 5 The title of the paper is provocative and interesting. Reading for pleasure has a number of advantages for physicians. The authors have described the process they followed for selecting the books and organizing the book clubs in detail. The limitations of the study have been well described. I have long been interested in reading outside of medicine. I must confess however, that my leisure reading activities have slowly declined. The benefits of doing so have been illustrated by the authors. In Nepal and the Caribbean, medical schools are standalone entities and the libraries usually have only medical books. Some of the medical schools at which I had worked had a good selection of fiction and other non-medical books. I and my colleagues use one-page excerpts from selected books during the health humanities module we facilitate. The Network toward Unity for Health conference organized in Kathmandu, Nepal was the only one among the many conferences I attended where there were activities related to the arts and the humanities. These are still not a part of conferences in the developing world. I would like to congratulate the authors on their initiatives and am hopeful that book clubs and other activities related to the humanities will become an integral part of medical conferences all over the world.

Reviewer Expertise:

NA

No decision status is available

# Reviewer report: Reviewer response for version 1

**DOI:** 10.21956/mep.19622.r29191

**Published:** 2018-07-18

**Authors:** Michaela Kelly

**Affiliations:** 1 University of Queensland

This review has been migrated. The reviewer awarded 4 stars out of 5 Thank you for this inspirational work. The benefits of engaging in non-medical reading are significant for health and well being and connecting with issues outside our sometimes somewhat confined existences. A Book club offers conference participants a chance to meet and understand others beyond what is normally achievable at a networking function. A great idea!

Reviewer Expertise:

NA

No decision status is available
